# Supplementary material for: Magnetically-driven colossal supercurrent enhancement in InAs nanowire Josephson junctions
Source: Nat Commun. 2017 Apr 12;8:14984. doi: 10.1038/ncomms14984 (PMC5394342; doi:10.1038/ncomms14984)
Supplement: Supplementary Information — Supplementary Figures, Supplementary Notes and Supplementary References [file ncomms14984-s1.pdf]

Supplementary Information

I. SUPPLEMENTARY FIGURES

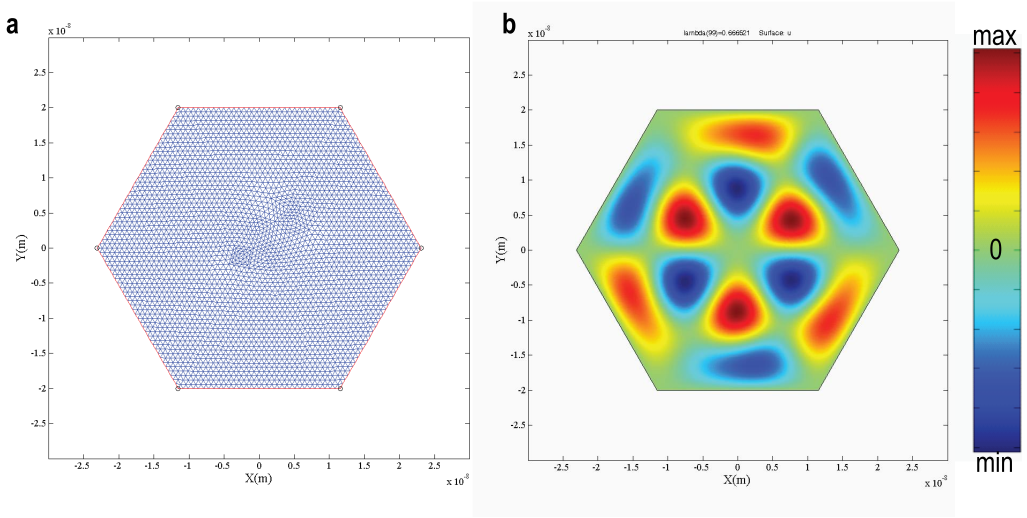

Supplementary Figure 1. | **Eigenmodes Calculation.** **a** Mesh structure used for the numerical solution of the Schroedinger eqution. **b** Colorplot of the wavefunction for transverse eigenstate number 19.

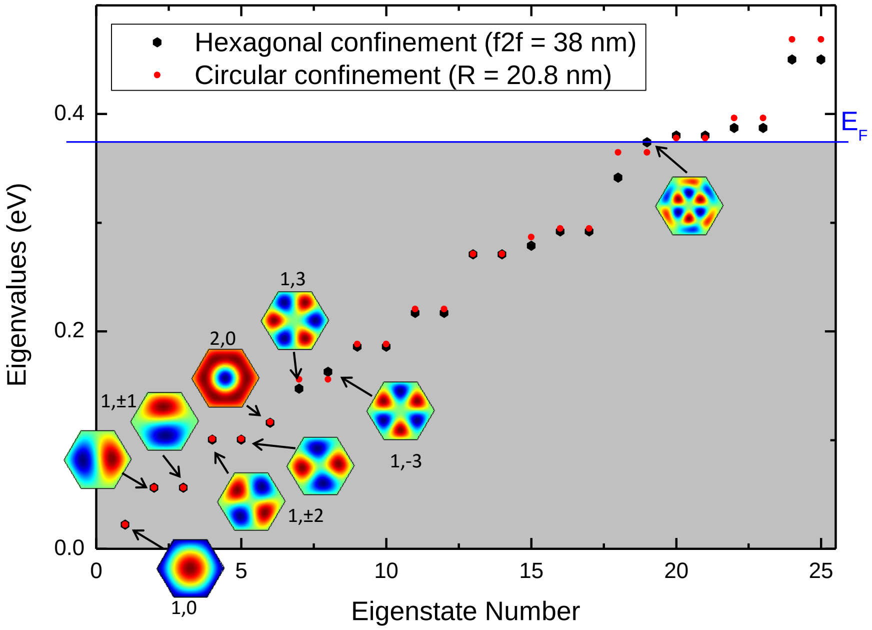

Supplementary Figure 2. | **Nanowire eigenenergies.** Energies for subband edges, computed for a haxagonal(circular) nanowire of 38 nm face-to-face (f2f) distance (20.8 nm radius (R)). Blue line marks the Fermi energy for density $3 \cdot 10^{18}\text{cm}^{-3}$ (19 occupied spinful subbands). Note that the last occupied subband lies very close to the Fermi energy.

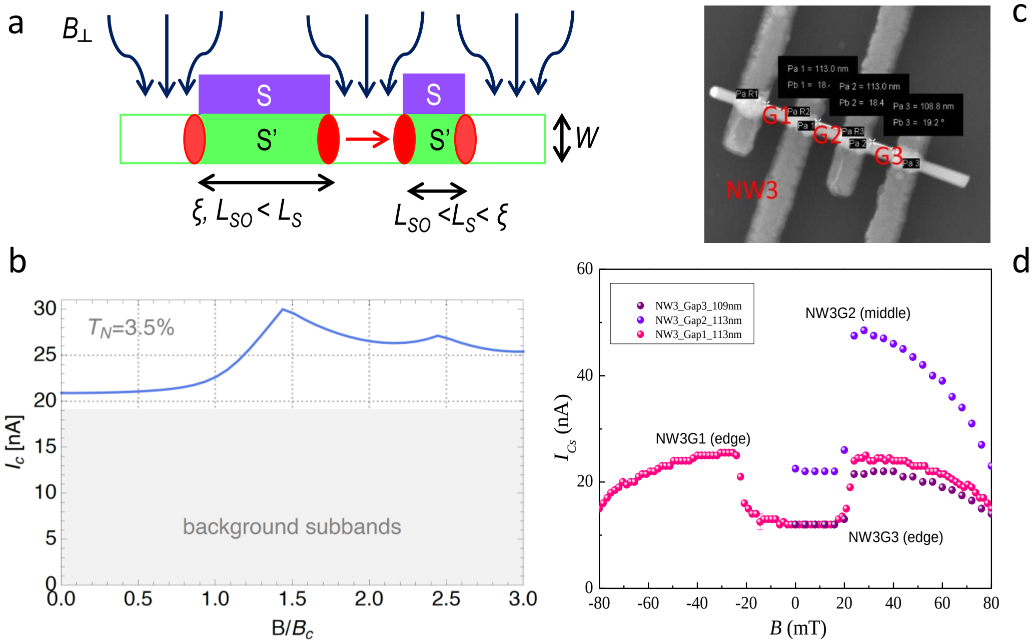

Supplementary Figure 3. | **Asymmetric Josephson junction a**, A sketch of the four Majorana bound states (red circles) formed in the two proximitized regions S' of a (*ballistic*) NW under the action of an out-of-plane magnetic field $B_\perp$. Here the length of the right superconducting lead is smaller than the size of the single Majorana's bound state (ξ) but still larger than the spin orbit length (L_{SO}). **b**, Theoretical critical current I_C calculated for increasing magnetic field values in an InAs Josephson junction similar to the ones investigated in the experiment ($L =$ 50 nm, L_S =100 nm and 500 nm, and $W = 40$ nm). The NW charge density $3 \cdot 10^{18}\mathrm{cm}^{-3}$ corresponds to 19 occupied spinful subbands. Rest of the parameters: spin-orbit coupling $\alpha_{SO} = 0.13\,\mathrm{eV\AA}$, contact transmissivity per mode $T_N = 53.\%$, and zero-temperature superconducting energy gap $\Delta = 200\,\mu\mathrm{eV}$. **c**, SEM image of one of the NW with three Josephson junctions of similar length. **d** Critical current vs out-of-plane magnetic field showing a more pronounced enhancement for the middle junction.

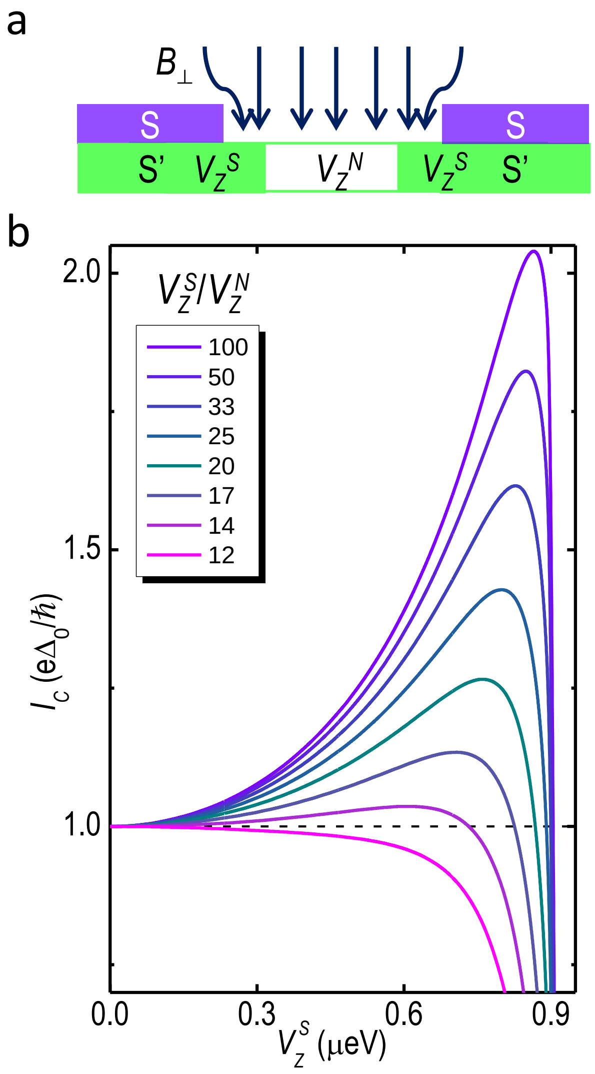

Supplementary Figure 4. | **Enanchement induced by an Inomogeneous Zeeman field.** **a**, Sketch of a (*diffusive*) NW Josephson junction in the presence of an inhomogeneous magnetic field due to the focusing effect induced by the two superconducting leads. The local Zeeman exchange energy in the superconductors S' and in the NW is denoted by V_Z^S and V_Z^N, respectively, with $V_Z^S > V_Z^N$. **b**, Theoretical critical current I_C calculated as a function of V_Z^S for different degrees of inhomogeneity (V_Z^S/V_Z^N).

II. SUPPLEMENTARY NOTES

1. Role of Asymmetries In our experiments, critical currents always exhibit, to some extent, an increase at a certain magnetic field, regardless of which (adjacent) pair of contacts is used. The most robust jumps, however, are usually observed in the contacts at the middle of the wire. At first glance, it might appear implausible that Majoranas can still contribute a sizeable supercurrent in the Josephson junctions formed the two side of the NW, as the hybridization of spatially overlapping Majoranas in the short superconducting lead ($L_S \simeq 150$ nm) should destroy the effect. However, a quantitative simulation shows that this is not the case when the system is close to the subband depletion $\mu_n \simeq 0$ (which is the scenario explored in our topological model). Indeed, the I_C enhancement is rather generic down to $L_S \gtrsim 100$ nm (i.e of the order of the spin-orbit length). As shown in Supplementary fig. 3b the enhancement in our simulations becomes gradually smaller as the junction is moved towards the edge of the wire, and the transition B is increased. The reason for the existence of these remnants of the topological transition and Majoranas is that, while outer and inner Majoranas on the short side of the junction overlap spatially, their energy splitting away from zero remains very small close to the transition at $B = B_C$, effectively mimicking a longer wire to some extent. This theoretical picture remains consistent with our experimental observations, which thus strengthens our topological interpretation. From the experimental point of view, the only device that allows a concrete comparison between edge and middle junctions is reported in Supplementary Fig. 3d In this device all the junctions have similar length ($L \simeq 110$ nm and $L_S \simeq 150$ nm) and we can notice a more pronounced enhancement in the middle one.

2. Alternative scenario -Inhomogeneous Zeeman field- If we exclude the topological transition scenario as the origin of the observed effect an alternative model that predicts an enhancement of I_C is based on a SF-I-SF Josephson junctions, where SF denotes a superconductor with a Zeeman-split density of states, and I is an insulator.

If the effective spin-splitting Zeeman fields in the left and right electrodes are different, $V_Z^{l(r)}$, one expects to observe an enhancement of the critical current with a maximum at $V_Z^l - V_Z^r = \Delta_l + \Delta_r$ [1, 2], where $\Delta_{l(r)}$ is the pairing potential in the left(right) superconductor. The enhancement of I_C occurs when the transmissivity of the barrier is low [2] to avoid the coupling between the two spin-polarized superconducting condensates. In addition, the critical current enhancement is also possible in SF-I-N-I-SF [3] and even in S-I-N-I-S junctions. In the latter case the enhancement of I_C occurs if the following conditions are fulfilled: (i) The density of the states of the S electrodes is BCS like with the coherent peaks Zeeman split thanks to the presence of the magnetic field (within this model the orbital effects are disregarded); (ii) The Zeeman fields in the S and N region ($V_Z^{S(N)}$) are very different in amplitude; specifically, it is required $V_Z^S \gg V_Z^N$ to achieve the observed I_C enhancement; (iii) the interface between the S electrode and the N wire should exhibit a low transmissivity. Condition (i) is plausible for the regions of the NW close to the S/N contacts, therefore one can identify the structure as a SF-I-N-I-SF, where now SF denotes a BCS superconductor with a variable spin-splitting proportional to the applied magnetic field. Condition (ii), namely an inhomogeneously distributed Zeeman field, is possible as well owing to the strong magnetic field focusing originating from the S electrodes so one can safely assume that $V_Z^S \neq V_Z^N S$ (see the scheme in Supplementary Fig. 4a). Condition (iii), on the other hand, is more unlikely but still possible by considering local defects in the NW.

Having in mind these limitations, we have computed the critical current of a diffusive SF-I-N-I-SF junction with the help of the quasiclassical Green's function formalism [3] (see next section for more details), and using the parameters obtained from the $I_C(T)$ fit reported in the main text. The results are summarized in Supplementary Fig. 4d. The overall behaviour is qualitatively consistent with the experimental observation although a quantitative comparison is difficult due to the complexity of the junction geometry. Despite the good qualitative description of $I_C(B)$, the main discrepancy between the model and the experiment lies in the large value of field inhomogeneity, $V_Z^S/V_Z^N \gtrsim 10$, required to obtain a magnetically-driven I_C enhancement. This last condition is indeed much stronger than the maximal expectation obtained for our junctions geometry in the out-of-plane magnetic field configuration, i.e., $V_Z^S/V_Z^N \lesssim 1.2$, as displayed in Supplementary Fig. 4b.

We have calculated the critical current I_C of a diffusive SF-I-F'-I-SF junction by solving the Usadel equation within the quasiclassical Green's functions formalism [3]. Above, SF denotes a superconductor (S') Zeeman split by an exchange field V_Z^S, F' is a N wire possessing an internal exchange energy V_Z^N, and I represent an insulating tunnel barrier (see the sketch shown in Supplementary Fig. 4a). I_C can be expressed as

$$I_C = \frac{\pi A k_B T}{e} \sum_{\omega} \left[\frac{(f^+)^2}{\sinh(\chi^+ L)\chi^+} + \frac{(f^-)^2}{\sinh(\chi^- L)\chi^-} \right], \quad (1)$$

where $\chi^{\pm} = \sqrt{2(|\omega| \pm i\,\mathrm{sign}[\omega V_Z^N])/D}$, $f^{\pm} = \Delta(V_Z^S)/\sqrt{(\omega \pm i V_Z^S)^2 + \Delta^2(V_Z^S)}$, the sum is over the Matsubara frequencies ω already introduced, and $\Delta(V_Z^S)$ is the effective superconducting order parameter calculated self-consistently from the BCS gap equation [4]. Moreover, $A = \frac{R_{NW} A_{NW}}{2 R_C^2 A_C^2 L}$, where R_{NW} is the resistance of the NW of length L, A_{NW} is the NW crossection, R_C is the contact resistance, and A_C is the contact area. For the calculation of the curves displayed in Supplementary Fig. 4b we have used the junction geometrical dimensions, the parameters obtained from the diffusive $I_C(T)$ fit shown in Fig. 1e of the main article,

and $T = 60$ mK.

[1] Bergeret, F. S., Volkov, A. F. & Efetov, K. B. Enhancement of the Josephson Current by an Exchange Field in Superconductor-Ferromagnet Structures. *Phys. Rev. Lett.* **86**, 3140–3143 (2001).
[2] Chtchelkatchev, N., Belzig, W. & Bruder, C. Josephson effect in SFXSF junctions. *JEPT Lett.* **75**, 646–650 (2002).
[3] Strambini, E., Bergeret, F. S. & Giazotto, F. Mesoscopic Josephson junctions with switchable current-phase relation. *EPL* **112**, 17013 (2015).
[4] Giazotto, F. & Taddei, F. Superconductors as spin sources for spintronics. *Phys. Rev. B* **77**, 132501 (2008).
